# Comprehensive Management of Multiple Dental Injuries After a Single Traumatic Event: A Case Report

**DOI:** 10.7759/cureus.90144

**Published:** 2025-08-15

**Authors:** Fahad M Alrabie, Mohammed A Alzubaidi, Muaath H Alzahrani, Bandar S Shukr, Sattam T Alammari, Anas A Alobaidi

**Affiliations:** 1 Faculty of Dentistry, Taif University, Taif, SAU; 2 Department of Preventive Dentistry, Faculty of Dentistry, Taif University, Taif, SAU

**Keywords:** crown fracture, crown-root fracture, delayed treatment, multidisciplinary approach, pediatric dental trauma

## Abstract

Traumatic injuries most commonly affect the maxillary teeth, making them highly susceptible to fractures. This study aims to report a case of multiple dental traumatic injuries after a fall, affecting maxillary central incisors, with a focus on their impact on the patient’s quality of life and the management approach that utilized a multidisciplinary team of dental specialists. A 12-year-old patient who presented approximately one and a half years after the initial trauma. The patient exhibited a complicated crown-root fracture with extrusion of the maxillary right central incisor, which was deemed non-restorable and managed through extraction and the placement of an immediate removable partial denture to restore aesthetics and function. The maxillary left central incisor sustained an uncomplicated crown fracture that progressed to pulpal necrosis due to prolonged dentin exposure; it was treated with non-surgical root canal therapy followed by a composite restoration. This case highlights the clinical challenges associated with delayed presentation and underscores the importance of early intervention and a multidisciplinary approach in achieving optimal functional and aesthetic outcomes in the management of dental trauma.

## Introduction

Traumatic dental injuries are mostly seen in children and adolescents, comprising 5% of all injuries. 25% of all school children experience dental injuries, and 33% of adults have experienced trauma to the permanent teeth, with most of the injuries occurring before the age of 19 years and affecting anterior teeth [1].

Dental traumatic injuries encompass a wide range of clinical presentations, each varying in prevalence, severity, and management complexity. A complicated crown-root fracture is relatively uncommon and is characterized by a fracture line extending from the crown into the root in an oblique direction [2]. When the fracture extends beyond one-third of the root length, the tooth is often deemed non-restorable, and extraction is generally indicated [3,4]. Extrusion, defined as partial displacement of the tooth out of its socket, requires immediate repositioning and stabilization to preserve periodontal ligament vitality and optimize prognosis [5]. An uncomplicated crown fracture, in contrast, involves only enamel and dentin without pulp exposure, and can be managed by fragment reattachment or composite restoration if treated promptly [5].

Delayed or inadequate management of such injuries can compromise prognosis, leading to pulpal necrosis, tooth loss, and more complex rehabilitative needs. This case report describes a 12-year-old patient who presented 18 months after a single traumatic incident with both complicated and uncomplicated crown fractures, as well as extrusion that affected the maxillary central incisors. The case highlights the impact of delayed intervention on prognosis, its effect on the patient’s quality of life, and the multidisciplinary approach required for optimal management.

## Case presentation

**Figure 1:**
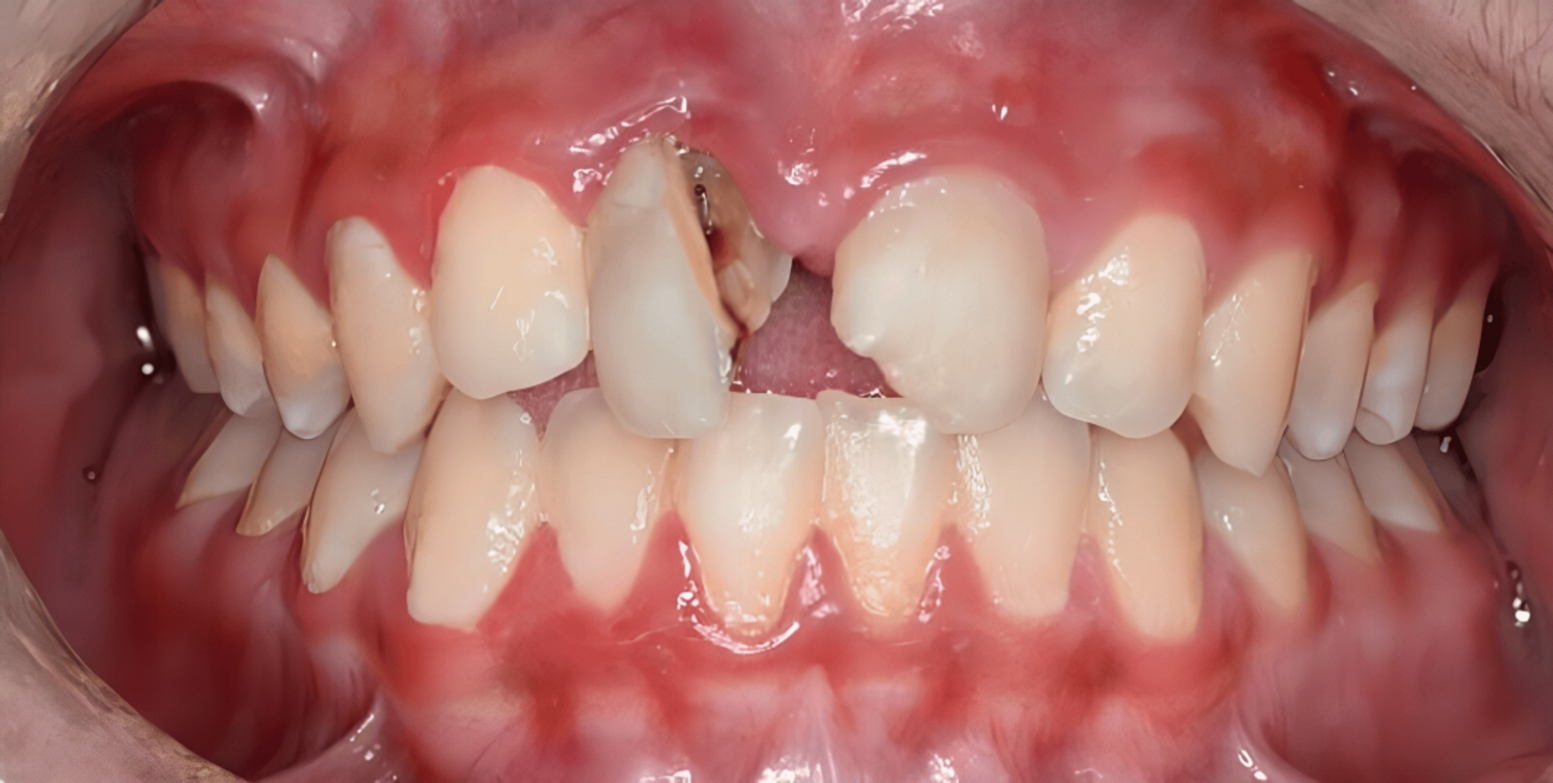
Pre-operative photograph.

**Figure 2:**
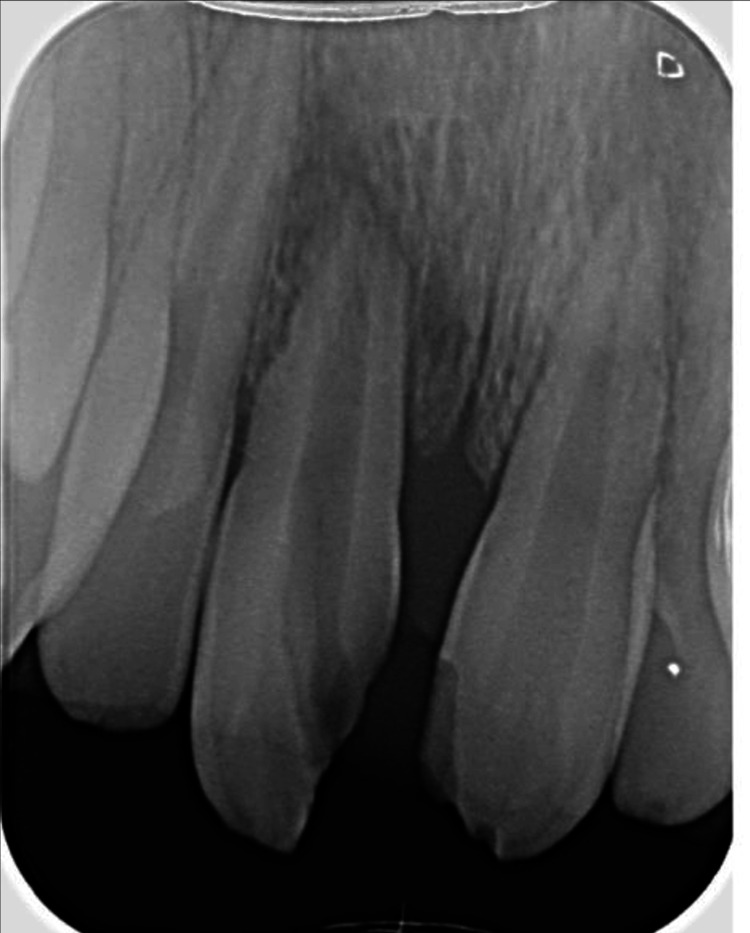
Pre-operative radiograph.

A healthy 12-year-old male attended Taif University Dental Hospital with his mother, seeking treatment for his upper front teeth. The mother reported that “my son has two upper frontal teeth with pain and mobility due to a dental injury.” The trauma occurred approximately one and a half years earlier when the patient fell forward onto rocky ground while climbing a mountain, striking his mouth directly. He experienced immediate pain, mild bleeding, and visible displacement of one of the upper central incisors. No professional dental intervention was undertaken at the time due to the family’s economic constraints and limited awareness of existing free dental care services. Over the following months, the patient experienced intermittent pain and progressive mobility of the right central incisor, as well as chipping of the left central incisor. These symptoms, along with aesthetic concerns and difficulty biting certain foods, ultimately prompted the family to seek dental care. The clinical examination revealed trauma to the maxillary central incisors. Pulp vitality was assessed using electric pulp testing and cold testing. The maxillary right central incisor exhibited an Ellis Class VI injury, characterized by a complicated crown-root fracture with extrusion and root involvement. The tooth was non-responsive to vitality tests, confirming pulp necrosis, and showed grade III mobility (Figure 1). The maxillary left central incisor initially sustained an Ellis Class II fracture involving enamel and dentin without pulp exposure. However, at the time of examination, the tooth tested non-responsive on vitality tests, indicating that pulpal necrosis had developed subsequent to the injury. This tooth also demonstrated grade II mobility. Radiographic examination revealed symptomatic periapical periodontitis affecting both teeth, with external inflammatory root resorption evident on the maxillary right central incisor (Figure 2).

**Figure 3:**
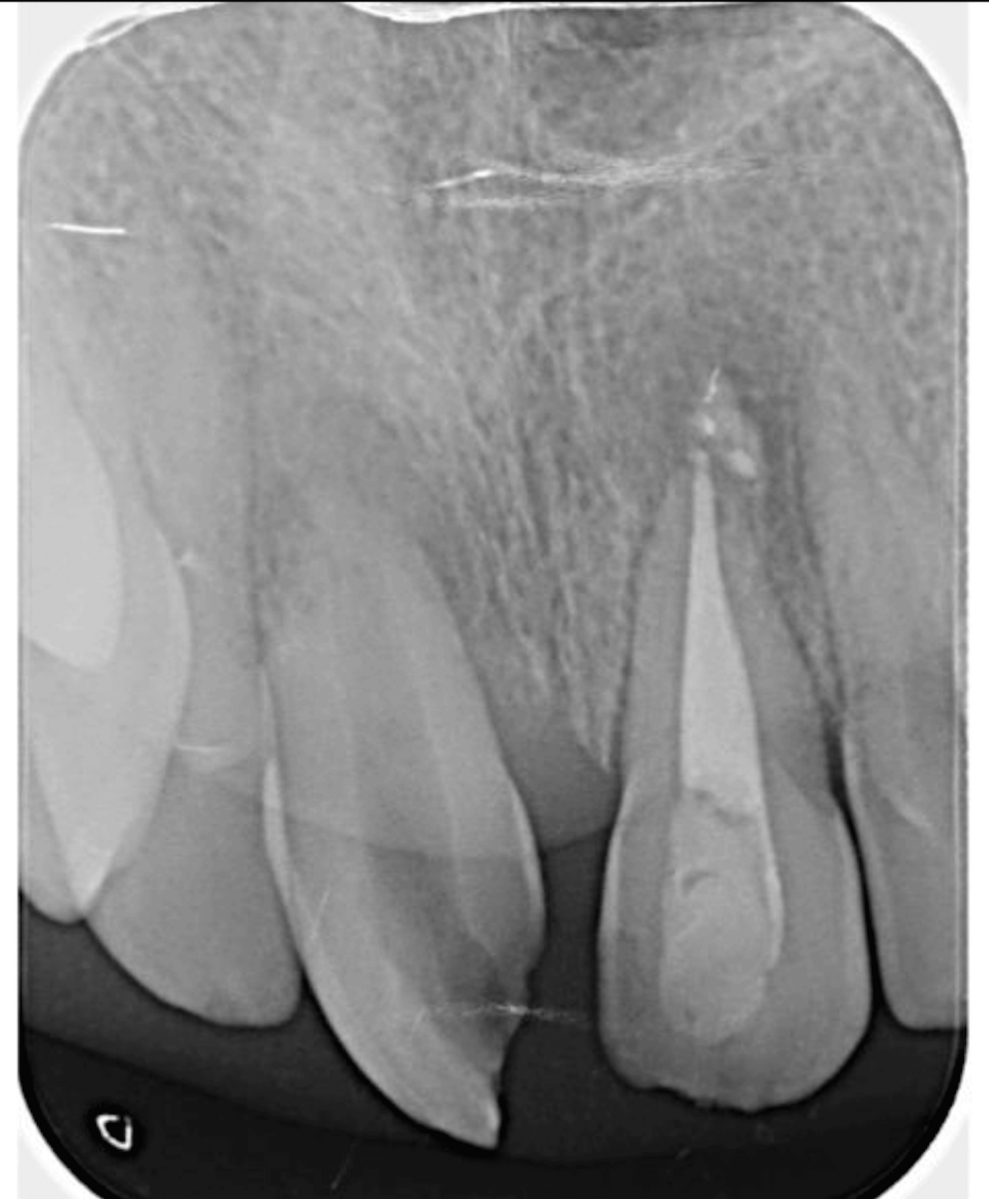
Postoperative radiograph.

After obtaining informed consent from the patient and his mother, non-surgical root canal treatment was initiated on the maxillary left central incisor. Following local anesthesia and rubber dam isolation, access cavity preparation was performed. Working length was determined using an apex locator and confirmed radiographically. Mechanical instrumentation was carried out using rotary files, with continuous irrigation of 2.5% sodium hypochlorite throughout the procedure. Calcium hydroxide was used as an intracanal medicament and placed inside the canal for two weeks to ensure disinfection and promote periapical healing. At the subsequent visit, the canal was thoroughly irrigated, dried, and obturated with gutta-percha using the cold lateral compaction technique. Finally, the tooth was restored with a composite resin restoration to restore function and aesthetics (Figure 3).

**Figure 4:**
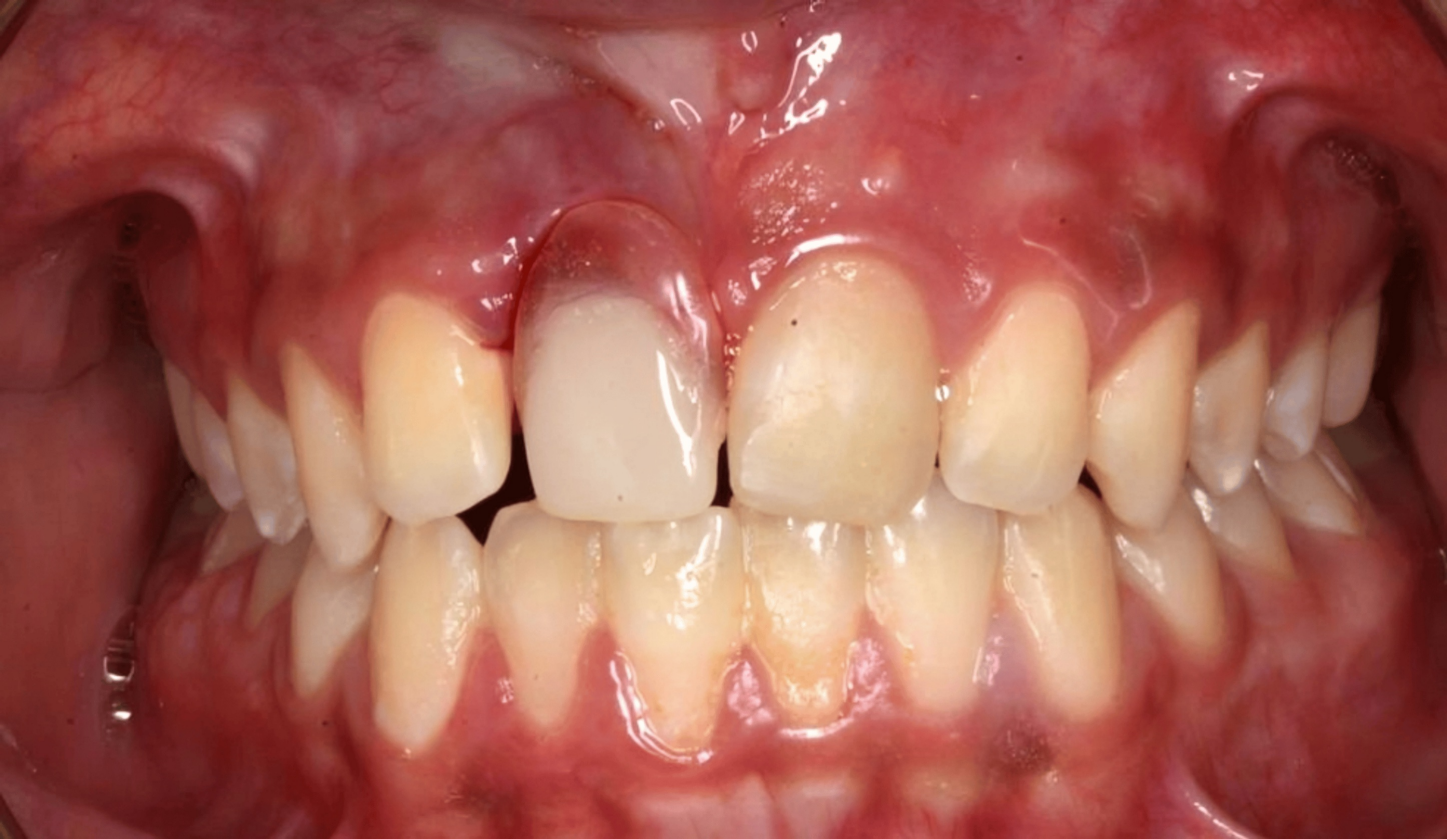
Postoperative photograph.

**Figure 5:**
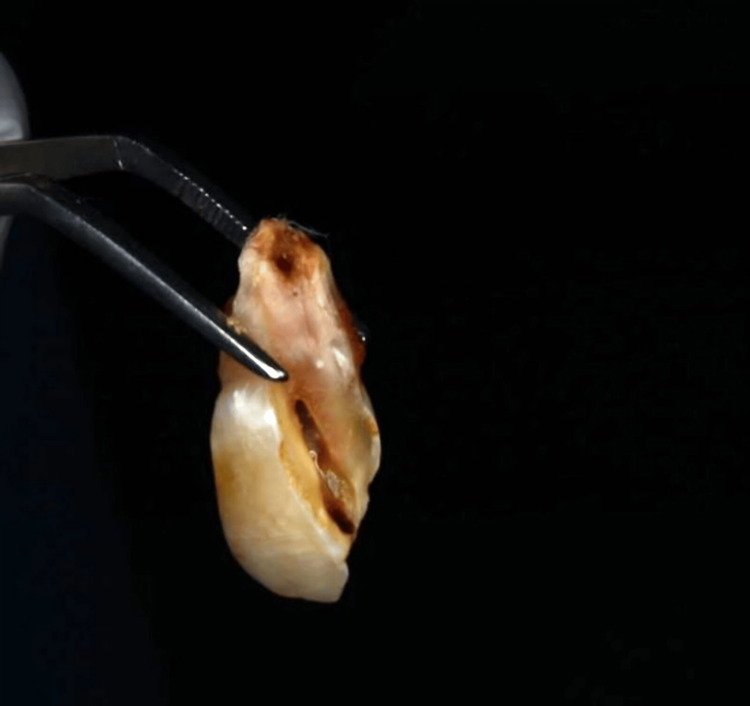
Extracted maxillary right central incisor.

The maxillary right central incisor was extracted as the fracture was extended to the root apically and went deep subgingivally, and cannot be restored in this situation, followed by the insertion of an immediate removable partial denture to restore aesthetics and function (Figures 4-5). This treatment option was thoroughly reviewed in consultation with the endodontist, prosthodontist, periodontist, and the restorative dentist, all of whom reached a consensus on its suitability. This treatment approach serves as a provisional solution until the completion of the patient’s growth, at which point definitive management may involve implant placement or orthodontic space closure accompanied by reshaping of the lateral incisors. An orthodontic evaluation was also conducted to assess any displacement or long-term complications, and no immediate orthodontic intervention was indicated at this stage.

The patient was placed on a regular follow-up protocol, with clinical and radiographic assessments conducted at three-month intervals. The clinical and radiographic evaluations demonstrated satisfactory healing of the left central incisor with no symptoms or progression of pathology. The removable partial denture was well tolerated and maintained function and aesthetics. These outcomes confirmed the effectiveness of the multidisciplinary treatment approach used in this case and highlighted the importance of ongoing monitoring and follow-ups in similar cases of complex dental injuries.

## Discussion

The management of crown or crown-root fractures in children’s incisors presents a considerable challenge for clinicians, as it necessitates the concurrent resolution of functional, aesthetic, and biological factors [6,7]. These injuries commonly result from falls, sports-related impacts, or accidents involving direct trauma to the face and mouth, especially in active children and adolescents [8,9]. Environmental factors such as climbing or playing on uneven terrain, as in our patient’s case, increase the risk of such injuries. A variety of treatment options for crown-root fractures are available based on the position, extent, and severity of the fracture [7]. These include crown lengthening to expose crown margins, orthodontic extrusion, intentional replantation through surgical repositioning, restorative techniques for managing subgingival margins, decoronation with root submergence, or extraction [10].

Untreated complicated crown-root fractures may lead to pulp necrosis, apical periodontitis, and root resorption [11]. Our case aligns with these findings, presenting pulp necrosis, apical periodontitis, and external inflammatory root resorption in the maxillary right central incisor, alongside tooth extrusion, all of which negatively influence prognosis. Similar cases in the literature report that complicated crown-root fractures often require extraction and provisional prosthetic rehabilitation, with definitive treatment delayed until growth completion [12,13]. These reports emphasize the importance of a multidisciplinary approach and the challenges posed by delayed presentation.

Following multidisciplinary discussion, extraction was deemed the inevitable treatment option, followed by insertion of a removable partial denture. An alternative for replacing a missing tooth in young patients is a resin-bonded bridge, which could be viable; however, it was not recommended in this case due to the mobility of the adjacent traumatized central incisor, as additional forces could worsen the prognosis. Definitive treatment options in the future may include orthodontic space closure with lateral incisor recontouring or dental implant placement post-growth completion [14].

Early intervention in cases of uncomplicated crown fractures is strongly advocated to ensure the preservation of pulp vitality in traumatized teeth [5]. Immediate sealing of exposed dentin is recommended to prevent bacterial penetration through dentinal tubules and to protect the pulp from potential thermal or chemical irritants that may lead to inflammation [13,15]. Management of fractured incisors may involve either reattachment of the fractured fragment or a composite resin restoration [5]. In this case, although the patient sustained an uncomplicated crown fracture, non-surgical root canal treatment followed by composite restoration was required due to prolonged exposure of unsealed dentin, which resulted in pulp necrosis.

## Conclusions

This case underscores the critical need for prompt and appropriate management of dental trauma to minimize complications and preserve both function and aesthetics. It also highlights the importance of a multidisciplinary approach involving pediatric dentists, endodontists, restorative dentists, prosthodontists, and possibly orthodontists to ensure comprehensive care tailored to the patient’s needs. Effective communication among the healthcare team, timely interventions, and individualized treatment planning can significantly improve outcomes. Moreover, long-term follow-up, which is applied in this case as part of routine dental care, is essential to monitor healing, detect potential sequelae, and support the child’s oral health development over time.
